# Challenges in Bone Tissue Regeneration: Stem Cell Therapy, Biofunctionality and Antimicrobial Properties of Novel Materials and Its Evolution

**DOI:** 10.3390/ijms22010192

**Published:** 2020-12-27

**Authors:** Oliver Riester, Max Borgolte, René Csuk, Hans-Peter Deigner

**Affiliations:** 1 Institute of Precision Medicine, Medical and Life Sciences Faculty, Furtwangen University, Jakob-Kienzle-Strasse 17, 78054 Villingen-Schwenningen, Germany; oliver.riester@hs-furtwangen.de (O.R.); box@hs-furtwangen.de (M.B.); 2 Institute of Organic Chemistry, Martin-Luther-University Halle-Wittenberg, Kurt-Mothes-Str. 2, 06120 Halle (Saale), Germany; rene.csuk@chemie.uni-halle.de; 3 EXIM Department, Fraunhofer Institute IZI, Leipzig, Schillingallee 68, 18057 Rostock, Germany; 4 Faculty of Science, University of Tuebingen, Auf der Morgenstelle 8, 72076 Tuebingen, Germany

**Keywords:** stem cell therapy, mesenchymal stem cells, antimicrobial materials, bone tissue engineering, critical large bone defects, high-throughput screening systems

## Abstract

An aging population leads to increasing demand for sustained quality of life with the aid of novel implants. Patients expect fast healing and few complications after surgery. Increased biofunctionality and antimicrobial behavior of implants, in combination with supportive stem cell therapy, can meet these expectations. Recent research in the field of bone implants and the implementation of autologous mesenchymal stem cells in the treatment of bone defects is outlined and evaluated in this review. The article highlights several advantages, limitations and advances for metal-, ceramic- and polymer-based implants and discusses the future need for high-throughput screening systems used in the evaluation of novel developed materials and stem cell therapies. Automated cell culture systems, microarray assays or microfluidic devices are required to efficiently analyze the increasing number of new materials and stem cell-assisted therapies. Approaches described in the literature to improve biocompatibility, biofunctionality and stem cell differentiation efficiencies of implants range from the design of drug-laden nanoparticles to chemical modification and the selection of materials that mimic the natural tissue. Combining suitable implants with mesenchymal stem cell treatment promises to shorten healing time and increase treatment success. Most research studies focus on creating antibacterial materials or modifying implants with antibacterial coatings in order to address the increasing number of complications after surgeries that are mostly caused by bacterial infections. Moreover, treatment of multiresistant pathogens will pose even bigger challenges in hospitals in the future, according to the World Health Organization (WHO). These antibacterial materials will help to reduce infections after surgery and the number of antibiotic treatments that contribute to the emergence of new multiresistant pathogens, whilst the antibacterial implants will help reduce the amount of antibiotics used in clinical treatment.

## 1. Introduction

Artificial biomedical implants have grown more complex because of progress in manufacturing processes as well as the creation of novel materials. The daily use of biomedical implants, including artificial organs, medical devices and disposable clinical apparatuses in clinical surgeries, has not only saved many lives but also restored the quality of life for many patients [1]. One field of application for biomedical implants is orthopedic surgeries, which have a market size of EUR 40 billion worldwide [2]. In particular, bone implants are used in the field of dental surgery as dental implants or bone void fillers. They are required after tooth loss as a result of disease or trauma, creating a need in the US market which has approximately 450,000 implants per year [3]. Another reason for the rising demand for bone implants is an increasing elderly population that requires treatment of various illnesses [4]. These illnesses include poorly healing large bone defects, which represent a major clinical problem [5]. The human body is not capable of regenerating critical large bone defects on its own [6]. Therefore, bone grafts must either be implanted with specific properties to induce bone growth or treated with stem cells to achieve regeneration of damaged tissue [7,8]. Even though implants have improved significantly over the last few decades, they still cannot replace original tissues without side effects. These side effects include irritation, inflammation and rejection of the implant. To date, autologous implants are the standard, but they bear the disadvantage of high complication rates of 10–40% and can cause hemorrhage, nerve and vascular lesions and postoperative pain [9].

New materials, in particular, must reduce negative host responses. Over the past few decades, interest in biodegradable materials has increased, as they eliminate the need to remove the implant, thereby reducing the number of surgeries required [10]. Biodegradable materials are also beneficial in accomplishing the ultimate goal of tissue engineering: the creation of tissue that is indistinguishable from natural tissue [11]. To achieve this, it is important to understand the effects of the used materials and their degradation products on the human body and to take these into account when developing new materials. Therefore, new materials need to be analyzed for cytotoxicity, antibacterial behavior, inflammatory responses, genotoxicity, cell attachment, tissue integration and metabolomic or proteomic changes of any involved cells [12,13]. Although materials used are tested for the listed parameters and are chosen to match the necessary mechanical and chemical properties to prevent complications, risks still remain.

Research efforts are aimed at novel alloplastic materials that have the potential to reduce high complication rates while being available in large quantities. They can be distinguished by their effects on the formation of new bone. Osteoconductive materials have the ability to act as a scaffold for the formation of new bone but do not have the ability to induce spontaneous osteogenesis when implanted subcutaneously, for example in mice [14]. Materials that can induce spontaneous osteogenesis are called osteoinductive. Novel synthetic materials must be osteoconductive to be suitable as a bone graft substitute, but they should preferably be osteoinductive to be used in critical large bone defects [15]. To achieve osteoinductivity, osteoconductive materials can be combined with proteins, such as bone morphogenetic proteins (BMPs), stimulating miRNAs, drug delivery systems containing small molecules or nanoparticles to induce osteogenesis [16,17,18,19,20].

Osteoinductive behavior is especially important when the implant is combined with stem cell treatment. Mesenchymal stem cells (MSCs) can differentiate into adipocytes [21], chondrocytes [22], neuron-like cells [23] or the osteoblastic lineage [7,24]. Their potential to increase the number of osteoblasts at the implantation site and their anti-inflammatory effects [25,26] make them the ideal candidates for supporting the bone healing process. MSCs can be obtained from the patient’s own tissues, such as adipose tissue, bone marrow or the umbilical cord [22,27], and subsequently expanded in vitro to generate the number of cells necessary for clinical treatment. By using autologous stem cells, complications such as implant rejection can be reduced to a minimum [28].

As mentioned above, some of the most serious risks for patients receiving an implant are infectious diseases, which contribute to 50–70% of the 2 million healthcare-associated infections in the USA [29]. As infectious diseases are a major challenge in current healthcare and especially for patients after surgery, implants with antibacterial effects would have two benefits: first, they can reduce the number of postoperative complications and, consequently, increase the overall chances of the treatment being successful; a second benefit would be a decrease in the amount of antibiotics needed, thereby reducing the number of emerging multiresistant pathogens. Every year, around 670,000 people in the EU are afflicted by an infectious disease with antibiotic-resistant pathogens; of these cases, 33,000 are fatal [30,31]. According to the World Health Organization (WHO), approximately 10 million global deaths will be caused by antibiotic-resistant infections in 2050 [32,33].

The optimal bone graft substitute must be a biodegradable, antimicrobial, biocompatible and osteoinductive material with favorable adhesive properties for MSCs. Since it is very unlikely that all of these properties will be found in one raw material, many approaches focus on modifying materials that have already shown promising results for some of these properties [2,34,35,36]. Modification can be done chemically by changing functional groups, by combining different materials or by loading the scaffold with carrier particles containing bioactive substances in order to obtain the desired properties [37,38,39]. As there are many novel materials and the number of samples to be analyzed continues to grow, screening systems for high throughput are becoming more important [40,41]. The bottleneck in the development of new types of implants is no longer the production of new materials, but the ability to effectively evaluate any modified materials [42]. Several approaches over the past decade have attempted to make high-throughput screening systems available to the majority of the research community [43,44]. These systems include microarrays, automated liquid handling systems and microfluidics. However, most of these systems are still very expensive, and efforts are being made to establish inexpensive and affordable processes [45,46]. For example, one of these processes is the production of microfluidic chips through additive manufacturing, which is also known as 3D printing technology [47,48]. A further improvement and availability of these screening systems will not only help in identifying the ideal candidate as a bone graft substitute, but also aid several other research areas such as stem cell differentiation, biomolecule- or cell-adhesion to surfaces or the effect of fluid dynamics on cell culture.

## 2. Bone Graft Substitutes for the Reconstruction of Large Bone Defects

**Figure 1:**
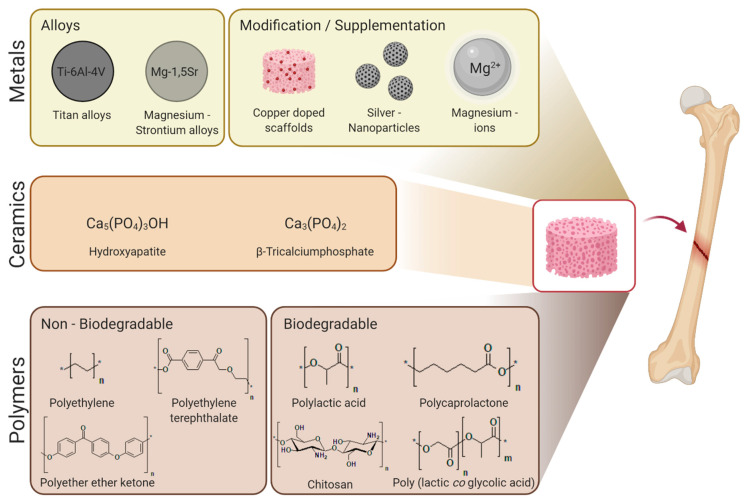
Material categories used in the creation of novel bone graft substitutes. Various materials that provide an alternative to autologous bone implants are available or in development. Metals have been used for the longest time because they are associated with mechanical stability and the manufacturing process is well known [49]. Biodegradable metal scaffolds made from magnesium alloys have shown promising results in bone regeneration and have recently attracted more attention [50,51,52,53]. Metal ions are used in the modification or supplementation of scaffolds to improve their regenerative or antimicrobial abilities as they have regulatory effects in cells [54,55,56]. Ceramics were seen as the most promising candidate because of their chemical similarities to natural bone [14,57]. However, since the manufacturing processes for ceramics cannot yet achieve the required mechanical stability, these bone grafts are only used for implants that are not mechanically stressed [58,59]. Polymers offer the greatest potential with an almost endless number of modification possibilities and adjustable manufacturing processes [60,61]. With increasing interest in biodegradable materials, the focus has shifted to polymers, as there are several different materials with known biocompatibility and biodegradation [62,63,64]. Figure created using app.biorender.com.

Recent publications dealing with large bone defects, the materials used in treatment and possible screening systems are evaluated in this review. The publications about materials can be divided into three main categories based on the materials used to produce new bone graft substitutes. These categories are metals, ceramics and polymers. Different materials used in studies to create novel bone grafts are shown in Figure 1. Each material has its advantages and disadvantages as a bone graft substitute, and their expected future potential consequently correlates with the numbers of recent publications. A detailed description of literature research with the keywords used and hits obtained is given in Table 1.

### 2.1. Metals

Based on their high mechanical stability and the existing knowledge of their biocompatibility, metals are currently irreplaceable for osteosynthesis of mechanically stressed bones [49]. However, most metals—which have been traditionally used as bone graft substitutes—are non-biodegradable [65,66], release toxic metal ions in vivo and have a high elastic modulus compared to natural bone tissue [56]. Recently, the biodegradability of the graft has become increasingly important, since the aim of tissue engineering is to restore a patient’s tissue with a material that is biologically adaptable. Wang et al. [65] and Li et al. [51] used a magnesium (Mg)–strontium (Sr) alloy (Mg and 1.5 wt% Sr) modified with micro-arc oxidation to produce bone grafts on the basis of its biodegradability and good mechanical properties. Strontium was added because of its capacity to induce bone formation and to prevent osteoporosis [51], according to Li et al. They demonstrated the cytocompatibility and osteogenic effects of the created Mg–Sr device in vitro with mouse bone-marrow-derived MSCs by showing upregulation of various osteogenic genes and cell proliferation similar to that of β-tricalcium phosphate (β-TCP) and calcium sulfate (CaSO_4_) grafts. Wang et al. observed the formation of new bone along ulna defects in vivo in New Zealand white rabbits using Mg–Sr alloy grafts compared to a control group treated with autologous morselized bone. Additionally, the authors observed biodegradation after immersion in Hank’s solution and in vivo resorption after implantation, which is a major advantage for magnesium-alloy-based bone grafts in comparison to biologically inert metal-based grafts. In contrast to biodegradable implants, biologically inert metal-based bone grafts have to be removed in a second surgery or must permanently remain in the patient. Both options can result in complications such as allergic reactions or secondary fractures [67,68]. Another approach uses the functional role of metal ions in the physiological and cellular environment to induce cell proliferation or osteogenic differentiation [54]. Based on the suggested approach, D’Mello et al. [55] observed bone healing of calvarial defects in rats using copper-loaded chitosan scaffolds. In their study, 2-fold and 11-fold higher ratios of bone volume to total defect volume were observed in comparison to chitosan scaffold or empty defect control groups, respectively. Zhang et al. [56] studied the effect of Mg ions on osteogenesis, chemotaxis and anti-alkaline stress in hFOB1.19 human osteoblast cells. They observed upregulation of osteogenic genes for the expression of Runx2 and alkaline phosphatase (ALP) through transient receptor potential melastatin-7/phosphoinositide-3-kinase signaling pathway. Furthermore, they observed upregulation of migration-associated factors like MMP2, MMP9 and vascular endothelial growth factor (VEGF) leading to osteoblast recruiting from low to high Mg ion environments. Xie et al. [69] developed a hybrid coating consisting of hydroxyapatite, silver nanoparticles and chitosan. The principal idea was to use the antibacterial effects of silver nanoparticles [70,71,72] in the creation of a coating for implants, leading to a decrease in postoperative infections. To counter the dose-dependent cytotoxicity of silver nanoparticles, Xie et al. [69] used chelatin and polydopamine as organic chelators to prevent the rapid release of silver ions from the coating. They showed the antibacterial effect of the developed coating to result in antibiofilm efficiency of 91.7%, 89.5% and 92.0% for *Staphylococcus aureus*, *Staphylococcus epidermidis* and *Escherichia coli*, respectively. Furthermore, they showed the formation of new bone in a longitudinal in vivo study by implanting HA/Ag/CS coated titan implants into rats. A scaffold-free approach—designed by Jia et al. [73]—used mesoporous silica-coated magnetic nanoparticles in distraction osteogenesis (DO) procedures for the treatment of large bone defects. Designed nanoparticles showed good in vitro biocompatibility for rat bone-marrow-derived mesenchymal stem cells and induced osteogenic differentiation. Results of quantitative reverse transcription polymerase chain reaction (qRT-PCR) indicated activation of Wnt/β-catenin signaling pathway, which plays an important role in osteogenic differentiation of the mesenchymal stem cells and in vivo bone formation [74,75,76]. Furthermore, Jia et al. [73] observed increased bone regeneration in a rat DO model analyzed by X-ray imaging, micro-CT, mechanical testing, histological examination and immunochemical analysis.

### 2.2. Ceramics

Ceramics are good implant materials given that their chemical properties are similar to natural bone tissue [14]. Furthermore, the most commonly used ceramics—calcium hydroxyapatite (HA) and β-tricalcium phosphate (β-TCP)—are biodegradable, osteoconductive and can be expanded by ceramic-based drug delivery systems to induce osteogenesis [77,78,79,80]. Consequently, in considering only the properties mentioned above, ceramics are the optimal material to create bone grafts similar to human natural bone. One reason why there still is no bone graft substitute that can perfectly replace natural bone is the immense importance of the bone graft structure for its mechanical and biological abilities [2]. To date, it is not possible to produce ceramic structures that have the mechanical stability of natural bone [58,81]. Furthermore, the handling of the material has to be reconsidered: either the geometry of the defect has to be adjusted to the geometry of the ceramic implants or the bone graft substitute has to be modeled to the desired shape. Modeling properties can be achieved by formulating a cement paste that can be injected directly into the bone defect or by creating an individual 3D-printed scaffold matching the bone defect geometry [82]. A downside of a cement paste is its lower mechanical properties [83]. Moreover, the high brittleness of cement paste and sintered ceramics when exposed to external forces are significant problems [84,85,86]. Recent research regarding ceramic-based bone grafts has therefore focused on modification of HA and β-TCP in order to increase the osteogeneration for bone grafts used in treatment of non-load-bearing bone defects. Xie et al. [57] studied the effect of strontium-doped calcium phosphate bone grafts in vitro on osteoblast-like ROS17/2.8 cells and in vivo on New Zealand white rabbits after implantation in bone defects. The results show improved biocompatibility and degradation properties for strontium-doped calcium phosphate grafts compared to calcium phosphate or hydroxyapatite grafts. Further immunohistochemical staining for VEGF indicated the potential of promoting angiogenesis. Tovar et al. [87] analyzed HA/β-TCP scaffolds of different compositions and evaluated them in a rabbit calvaria model, showing that all analyzed compositions were biocompatible and osteoconductive. The highest amount of new bone regeneration and the lowest amount of soft tissue infiltration were observed by using a composition ratio of 55% β-TCP and 45% HA. Weng et al. [16] assessed whether the addition of cinnamaldehyde has a positive effect on bone regeneration when combined with β-TCP scaffolds. Cinnamaldehyde is isolated from *Cinnamomum cassia* and has been reported to show anabolic effects on osteoblasts [88,89]. After implantation into critical-size calvarial defects (5 mm) in ovariectomized rats, Weng et al. showed an additive effect of cinnamaldehyde and β-TCP resulting in increased bone growth compared to a blank control or β-TCP scaffolds after 12 weeks of treatment. The positive effect of adding miRNA molecules to the scaffold was demonstrated by Hu et al. [20], whereby an improved tissue regeneration was achieved. Supplementation of miRNAs offers the possibility to regulate multiple signaling pathways without altering mechanical properties of the scaffold. Hu et al. identified an miRNA (miR-210-3p) that upregulates the expression of several osteogenic genes in vitro. They loaded a poly-L-lactic acid and a β-TCP scaffold with miR-210-3p and cultivated the scaffolds with bone-marrow-derived mesenchymal stem cells. The formation of new bone was observed after subcutaneous implantation in nude mice, implying osteoinductive properties. Furthermore, they showed significant repair of a canine load-bearing mandible bone defect using the miR-210-3p-loaded β-TCP scaffold when compared to the blank control or the β-TCP scaffold.

### 2.3. Polymers

Polymers have received comparatively more attention recently, since most metals have the crucial disadvantage of being biologically inert, whilst ceramics have extremely poor mechanical stability. Moreover, polymers offer several options for chemical functionalization that are useful in bone tissue engineering. This allows the behavior of the material to be individualized in order to adapt to the application, for example by adjusting biodegradability, antibacterial behavior, osteogenic properties or mechanical strength [60]. Possible modifications available for polymers include copolymerization, the creation of polymer blends or special geometries, chemical modification of monomers or the incorporation of inorganic nanocomposites into polymers [37,38,90]. Polymers are often categorized by their origin into synthetic polymers—such as polylactic acid (PLA), polyethylene (PE), polyglycolic acid (PGA), polycaprolactone (PCL) or poly (lactic-*co*-glycolic acid) (PLGA)—and natural polymers, such as collagen, gelatin, silk fibroin, chitosan, alginate or hyaluronic acid. A more suitable categorization is to evaluate the polymers from the perspective of the application, not the manufacturing. In this regard, the specification of biodegradability has become more attractive over the last few decades, particularly for implants that would otherwise have to be removed in a second surgery. The discussion in the following subsections is presented separately for non-biodegradable and biodegradable polymers.

#### 2.3.1. Non-Biodegradable Polymers

The bone tissue research community is not primarily focused on non-biodegradable polymers, as noted above. However, some approaches with non-biodegradable polymers show promising results in the antimicrobial modification of surfaces in order to address future problems with implant infections. For example, Rossetti et al. [91] obtained a polymer with antibacterial behavior for up to one month against *S*. *aureus*, *E*. *coli* and *Pseudomonas aeruginosa* by melt-compounding PE with quaternary ammonium salts. They showed the antibacterial effect after incubation overnight on the modified PE samples by means of live/dead staining. A comparable result was achieved by incorporating an N-halamine functionality into polyethylene terephthalate (PET) via Knoevenagel condensation in the polyester chain. The obtained polymers showed superior antibacterial activity with 100% reduction against *S*. *aureus* and *E*. *coli* within 30 min of incubation [92].

Poly (ether ether) ketone (PEEK), which is a widely used material for bone implants, was photochemically modified by utilizing a “grafting to” approach by cationic, quaternary ammonium containing methacrylate polymer brushes, influencing cell–surface interaction [93]. The material had an anti-adhesive effect against *E*. *coli*, resulting in less than 1% of the bacteria adhering to the samples. In a similar study, Ishihara et al. [60] modified PEEK substrates with different methacrylate layers and analyzed their behavior with regard to cell adhesion. A nearly 2-fold increase of adhered L929 fibroblast cells on a cationic modified surface in comparison to the control PEEK surface was observed in this study.

#### 2.3.2. Biodegradable Polymers

Many articles have been published in the field of biodegradable polymers considering their potential use as bone implant materials. Biodegradable polymers, such as PLA, are thermoplastic, allowing their usage as filaments in the process of additive manufacturing, commonly known as 3D printing [62,94]. This leads to the possibility of adapting the geometry of the bone implant to the defect of the patient, resulting in better ingrowth and healing [95]. More important factors for bone implants are the pore size and the pore shape of the scaffold, which are both necessary for optimal osteoblast proliferation [96,97]. The parameters mentioned can be adjusted in the fused deposition modeling (FDM) 3D-printing manufacturing process. The material biocompatibility of 3D-printed PLA in combination with hydroxyapatite and silk fibroin was demonstrated in vivo for the use as bone clips by Yeon et al. [98]. Polymer-based implants can also be improved by producing polymer blends with increased biocompatibility and biofunctionality compared to each material separately, for example by mixing PLA with PCL or hydroxyapatite. The mentioned materials were indirectly 3D-printed in different combinations by Hassanajili et al. [99] and tested for biocompatibility, osteoinductive behavior and mechanical strength in comparison to human bone tissue. An optimized blend with a weight ratio of 70/30 PLA/PCL was found to be the best for osteoblast functionality, indicated by increased ALP activity. Similar to the studies described for non-biodegradable polymers, Kalelkar et al. [100] used a click chemistry approach, modifying an azido-terminated PLA with different quaternary ammonium groups and creating a polymer showing antimicrobial activity against *E*. *coli* and *S*. *aureus*. The amino-functionalized polymer killed the cultured bacteria within 1 h of surface contact. Sharma et al. [17] used PLA in a copolymer with PCL cast into a porous scaffold. They implanted adenoviral infected PLA/PCL scaffolds seeded with rat bone-marrow-derived mesenchymal stem cells into a rat calvarial defect model and performed micro-CT analysis after 8 weeks of treatment. Results indicated that delivery of BMP2 alone showed better bone regeneration than BMP2 and VEGF together with 43.37 ± 3.55% and 27.86 ± 2.89% defect closure, respectively. These results were supported by histological and molecular analysis. Sharma et al. assumed that adenoviral delivery of VEGF inhibits the expression of BMP2 when coexpressed, as observed by Schönmeyr et al. [101].

Chitosan is another very popular polymer often used in the design of novel biodegradable implants because of its good biocompatibility and antibacterial behavior [102]. Yang et al. [103] designed a scaffold with antibacterial and osteogenic potential by combining 3D-printed PLGA/HA with a covalent hydroxypropyltrimethyl ammonium chloride chitosan (HACC) coating. Bone-repairing and antibacterial effects were studied in infected rat femoral shaft defect model and rabbit condyle defect model using X-ray, micro-CT, microbiological and histopathological analysis. They observed increased anti-infection and bone-healing capabilities for HACC-grafted PLGA/HA scaffolds in comparison to control groups and suggested treatment of infected bone defects with a combination of antibiotic scaffolds and systemic antibiotics in order to decrease the risk of antibiotic resistance. Whilst the mechanism of antimicrobial action is still unclear, it is generally assumed that it is due to the cationic charge of the glucosamine amino group leading to a disruption of the bacterial cell wall and leakage of cytosol [104,105]. However, Raafat et al. [106] found few similarities in transcriptional response patterns of chitosan compared to antimicrobial peptides that permeate the cell membrane and result in cell lysis [107,108], suggesting a different underlying antimicrobial mechanism of chitosan apart from its cationic properties. According to transcriptional analysis, chitosan treatment of *S*. *aureus* interacts with its metabolism by downregulating genes responsible for energy metabolism. Transmission electron microscopy imaging showed an intact but malfunctioning cell wall, leading to cytosol leakage without actual pore formation events [106]. The cationic character of chitosan is also one reason for its biocompatible properties, since the free amino group becomes easily protonated under physiological conditions, leading to complex formation with many negatively charged biomolecules, such as nucleic acids, proteins, growth factors and cytokines [109]. In addition, negatively charged glycosaminoglycans can bind to the material as a component of the extracellular matrix [110], leading to the recruitment of these proteins from the physiological environment to the implant surface. This produces a locally increased protein concentration and improved activity in relation to the surrounding tissue [111]. Chitosan can be modified by adding several functional groups, for example via phosphorylation for enhanced osteogenic differentiation and mineralization of bone tissue [112,113]. Sulfated chitosan has been shown to promote bone tissue formation and mineralization as well as vascularization of 3D-printed tissues in a HUVEC-MC3T3 cell coculture system. Interestingly, the osteogenic and vascular effects of sulfated chitosan were improved compared to phosphorylated chitosan coatings, as demonstrated by higher related gene and protein expression [114]. Soares et al. [115] created porous chitosan scaffolds via a freeze-drying process that involved blending chitosan with inorganic hydroxyapatite or other calcium salts. They adjusted the porosity of the scaffolds by altering the ratio of Ca(OH)_2_ suspension to chitosan during the freeze-drying process. The obtained construct released Ca^2+^ ions for up to 21 days. Human dental pulp cells were able to grow into the scaffold and showed improved metabolic activity towards calcium-rich matrix deposition. A porous chitosan scaffold was also obtained using ionic liquids, leading to swelling of water-insoluble chitosan to finally obtain a sponge-like, porous structure for cell encapsulation. The material displayed good biocompatibility towards human fibroblasts and human adipose stem cells [116]. Other natural polyaminoglycans, like gelatin [117], alginate [118] or a combination thereof [119], are used in the design of novel materials for bone grafts, such as the one designed by Lohmann et al. [120]. They developed an ArcGel based scaffold consisting of gelatin and PEO-PPO-PEO triblock copolymer (Pluronic F-108). The ArcGel was compared to standard autologous and commercially available Bio-Oss collagen implants using µ-positron emission tomography (µ-PET) to monitor bone healing and metabolic processes over treatment time.

The results show similar bone healing of osteoinductive ArcGel and autologous implants resulting in the creation of new bone hardly distinguishable from original bone tissue. In contrast, the Bio-Oss implant as an osteoconductive implant resulted in slower bone healing and was incorporated into the defect rather than remodeled into new bone.

Polymeric micelles can be loaded with peptides or drugs in order to supplement existing scaffolds with bioactive molecules. Capretto et al. [121] created polymeric micelles consisting of amphiphilic block copolymer Pluronic F127 in a continuous flow microfluidic device using a hydrodynamic focusing flow configuration. Micelles, including dexamethasone and ascorbic acid 6-palmitate, dissolved in DMSO, were tested for effects on the osteogenic differentiation of human periodontal ligament mesenchymal stem cells. The effect of drug-loaded polymeric micelles on osteogenic differentiation of mesenchymal stem cells was analyzed microscopically with alizarin staining of extracellular calcium deposits. Stem cells cultured with drug-containing micelles showed increased calcium deposits compared to stem cells cultured in standard culture medium supplemented with comparable concentration of dexamethasone and ascorbic acid. Polymeric micelles can also be used to transport other therapeutics, such as Gli inhibitors for the inhibition of tumor-induced bone disease as shown by Vanderburgh et al. [122], or to deliver functional proteins as demonstrated by Kallar et al. [123].

Some of the publications described used composite scaffolds because a combination of different materials such as ceramics and polymers and coating of scaffolds can achieve better results by taking advantage of the properties of each material. However, characteristics of heterogeneous composite scaffolds must be analyzed regarding biodegradation, since the interaction between the implant and the patient’s body takes place on the surface of the implant. The surface of the implant can change during treatment as a result of degradation and subsequent exposure of lower layers [2,124,125]. To evaluate biodegradation behavior or implant performance, the model must be carefully selected, particularly as the cell line or animal used can influence the results obtained, especially in in vitro models.

## 3. Stem Cell Treatment for Critical Large Bone Defects

**Figure 2:**
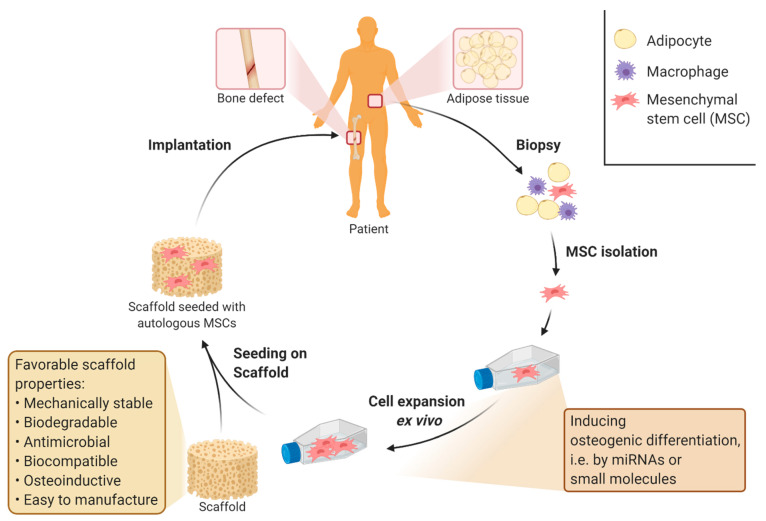
Treatment of large bone defects with bone implants supported by autologous mesenchymal stem cells. Application of mesenchymal stem cells (MSCs) in the treatment of large bone defects has been shown to reduce treatment time and increase the overall success rate of bone implants [137,138]. The number of cells required for clinical treatment can be obtained by ex vivo expansion of autologous stem cells derived from different tissues [127,139,140]. Suitable tissues for the isolation of MSCs are adipose tissue, bone marrow or umbilical cord [22,27]. During ex vivo expansion, osteogenic differentiation of MSCs can be induced, for example, by supplementing with specific miRNAs [132,133,134] or small molecules such as dexamethasone [135,136]. Figure created using app.biorender.com.

Many of the previously described studies used MSCs for showing in vitro biocompatibility and biofunctionality of designed materials or scaffolds [17,20,51,73]. MSCs are preferred because they are seen as the most promising candidate for supporting the healing process of implants at the cellular level and include the in vitro model that most closely resembles the natural bone healing process [126]. Reasons for this are their multilineage differentiation [7,127] and anti-inflammatory behavior [25]. Even though MSCs are relatively easy to obtain from the patient’s adipose tissue [126,128], the cell number isolated from the tissue is too low for direct use in clinical treatment. Therefore, stem cells have to be expanded ex vivo after isolation [129]. Subsequently to the expansion, stem cells can be seeded on the prepared scaffold with osteoinductive properties. These properties act as a signal for the MSCs to differentiate into osteoblasts so that the initial osteoblasts cell count in the scaffold is as high as possible [130,131]. Differentiation into the osteoblastic lineage could otherwise also be induced during ex vivo expansion by supplementing with specific miRNAs [132,133,134] or small molecules, such as dexamethasone [135,136]. A higher initial cell count of osteoblasts and MSCs at the implantation site is beneficial for the healing process and treatment time, as it drastically shortens the initial phase of cell migration and cell proliferation in the natural bone healing process [24]. A potential clinical application of autologous MSCs in the treatment of critical large bone defects is shown in Figure 2.

## 4. Test Systems in Material Evaluation

**Figure 3:**
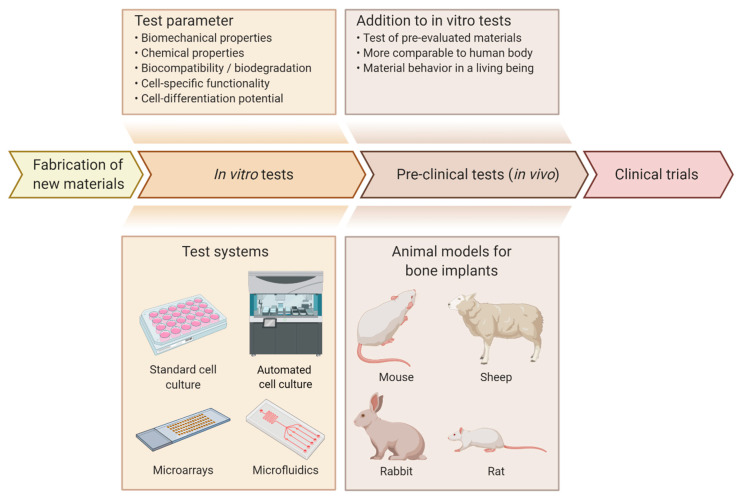
Overview of the evaluation process for novel materials/implants. After development and fabrication, materials are analyzed and assessed for their suitability for use in implants. In order to reduce the number of in vivo tests required, the materials are first tested in vitro [144]. In a first step, biomechanical and chemical properties as well as biocompatibility, biodegradation, cell-specific functionality and cell-differentiation potential are analyzed using in vitro models [18,57,122]. Various test systems are available for in vitro models, including standard cell culture, automated cell culture, microarrays and microfluidics. The advantages, limitations and applications of these systems are listed in Table 2. After the initial evaluation of the materials or implants, promising candidates are tested in in vivo models to obtain results that are more comparable to the human body and have a variety of biological mechanisms that are not implemented in in vitro models [141,142]. Animal models suitable for testing bone implants include mouse [145,146], rat [15,147], rabbit [57,148] and sheep [149,150]. Figure created using app.biorender.com.

Test systems, which can be used to assess the osteogenic potential of materials, can be categorized into in vivo and in vitro systems. In vivo test systems are the more accurate and complex systems of the two and reflect the subsequent application of the implant as close as possible [141,142]. Therefore, they are used in preclinical trials to assess the biofunctionality and biocompatibility of an implant in a living body with many complex systems influencing each other. This complexity has not been achieved in in vitro models so far [143]. Although in vivo studies are the more comparable model to the human body, they are usually avoided because of several downsides such as ethical issues, different cellular behavior between humans and animals and high costs caused by the need for highly qualified personal and suitable facilities for animal treatment. Consequently, in vitro models are often used to assess the suitability of a newly created material for an application. The results obtained can aid in the pre-evaluation of samples, thereby reducing the number of subsequent in vivo tests required [144], as shown in Figure 3.

There are a variety of cell lines commercially available, with distinct advantages and disadvantages that should be considered for each test model before establishing it. For example, if mineralization should be observed as an indicator for bone generation, a well-characterized osteoblast cell line such as Saos-2 or MG63 can be used [151,152]. These are immortal cell lines isolated from osteosarcomas [151,153] and have the advantage of being an established part of research studies and, consequently, well characterized and available in large quantities. The downside of immortal cell lines isolated from tumors is that they often behave differently to primary cells [153,154,155]. Therefore, many research studies, particularly those aimed at clinical approaches, use primary cells isolated from human or animal tissue. Their use gives the model better comparability with the natural tissue. However, the availability of primary cells is limited. Furthermore, primary cells may vary amongst donors, thereby making statistical analysis crucial [156,157,158] in order to exclude donor to donor variation. As mentioned, a model as close as possible to the human body is preferred to evaluate the performance of an implant in preclinical studies [144,159]. Outcomes may differ between in vitro and in vivo experiments because many biological factors are not fully understood yet and are missing in some in vitro experiments. Therefore, a combination of both models is recommended [142,143]. Nevertheless, in vitro models are excellent for understanding an underlying mechanism or for analyzing a specific behavior because they are easy to manipulate and comprehend. Conversely, in vitro models are the standard for the initial evaluation of new materials or drugs [13,160] based on cost-efficiency, ethical considerations and the possibility of high-throughput screenings. The efficient screening of materials becomes more important as the number of variables to be analyzed increases with implants becoming more complex. Implant performance can be influenced by several factors such as the ratio of used materials, the density of functional groups, the implant topography and surface degeneration or the modification with medication [161,162,163,164,165]. If the parameters influence each other and cannot be analyzed separately, the number of samples to be tested can quickly number into the thousands, making high-throughput assays essential for effective analysis. In vivo tests are not suitable for high-throughput assays since the number of animals required to perform these tests would be neither manageable nor ethically justifiable [166].

### 4.1. High-Throughput Screening Systems: Automated Cell Culture

Several methods are suitable for setting up a high-throughput screening system, as shown in Figure 3. The most obvious method is to scale up standard culture procedures and devices in quantity. However, the challenge here is that the experiments still need to be manageable, which is only possible if some of the most time-consuming processes, such as mixing and pipetting reagents, are automated. Such a semiautomated experimental design for evaluating the influence of small molecules on osteogenesis was described by Mazaki et al. [167]. The effects of 768 natural compounds from the RIKEN NPDepo library were analyzed by incubating the chemicals with immortalized bone-marrow-derived human MSC line UE6E7T-12 in 96-well plates. They identified a natural flavonol, namely kaempferol, with the ability to increase osteogenic markers and further compared it to ipriflavone, which has been described to be useful against osteoporosis [168]. More specifically, incubation with 35 µM kaempferol increased ALP activity by approximately 4.5-fold compared to the control group. On the other hand, the addition of 35 µM ipriflavone had no significant effect on ALP activity. A similar study was conducted by Brey et al. [136] to study the effect of small molecules on the osteogenic differentiation of MSCs. Brey et al. analyzed the effects of 1040 small molecules of the National Institute of Neurological Disorders and Stroke chemical library on MSCs and identified 36 promoters and 20 inhibitors of osteogenic differentiation. Cells were cultured in 384-well plates with 10 µM soluble factors of the library and 0.1% DMSO. After 8 days, cell viability and osteogenesis were analyzed with AlamarBlue assay and alkaline phosphatase assay, respectively. Addition of the 1040 soluble factors was performed using a robotic liquid handling system, which is not part of standard equipment in most laboratories and is, therefore, a limitation for quickly imitating the described method [169,170].

The formation of new bone tissue depends not only on the proliferation of functional osteoblasts but also on other important factors, such as the revascularization of newly formed tissue or biodegradation of the implant [171]. The perfect material should degrade as fast as the patients’ tissue grows, resulting in a replacement of the implant by functional tissue. Tzeranis et al. [172] developed a high-throughput degradation monitoring device to analyze the biodegradation of different tissue engineering constructs. The device monitors the deformation of the construct under static gravity load correlated with its degradation. Device capabilities have been shown by the degradation of fresh human cartilage samples treated with 0.5 or 1 mg/mL collagenase over 3 days resulting in 2.77 ± 0.52% and 6.39 ± 0.99% strain, respectively. The authors mentioned that the device can also be used to quantify the effects “on the remodeling of various kinds of tissues, implants and tissue engineering constructs” [172].

### 4.2. High-Throughput Screening Systems: Microarray Devices

Microarray applications have the potential to screen large libraries of chemicals and are not limited by a predefined number of wells. The only limitation on the quantity of samples is defined by the accuracy of the microarray printer and the chip size. For this method, the materials to be analyzed need to be printable, such as monomer solutions or dissolved polymers, or have to be immobilized on the chip’s surface, such as proteins or DNA. A microarray library with 496 different polymers reported by Mei et al. [173] was printed on a conventional 25 x 75 mm glass slide and subsequently polymerized via exposure to UV light. Polymers were coated with fibronectin to increase cell adhesion. The microarray was designed to study polymer surfaces for islet cell cultures by evaluating cell adhesion and insulin expression on different synthetic polymers. As a result, Mei et al. identified a polymer showing cell attachment for Sprague Dawley rat islet cells such as coated cell culture polystyrene dishes, which are standard in islet cell cultivation. Furthermore, they showed a different attachment behavior for human embryonic stem cells, highlighting the potential of microarray applications in the identification of cell type specific interactions, for example for MSCs on the implant surface, or antibacterial behavior, as suggested by Khan et al. and Jiang et al. [174,175]. Valles et al. [164] designed a microarray printer, which photochemically immobilizes glycan molecules on thiol-terminated surfaces for the analysis of lectin-glycan binding interactions. Combinations of eight lectin solutions and five different glycosides of varied densities were generated using a microfluidic chip with chaotic mixers. Valles et al. designed a microarray chip with 100-fold smaller features than conventional methods and monitored the kinetics of five glycans by fluorescent labeling by combining microfluidics with the computer-controlled activation of photochemical chemistry. The proof of concept study showed the potential of this method in high-throughput screenings of binding kinetics for use in the analysis of novel therapeutics or material interactions.

### 4.3. High-Throughput Screening Systems: Microfluidic Devices

Although microarrays are suitable for handling immobilized materials, they are limited in fluid studies (see Table 2). In this case, microfluidic devices show their strengths by offering the possibility to control different fluids on a chip. They have the potential to model a cellular environment more similar to the natural one than the one achieved in static cultures [176]. Additionally, the possibility to adjust the perfusion rate of cell chambers can be a huge benefit, as for example in models analyzing vascularization. As previously mentioned, vascularization is just as important to the success of the implant as cell-specific functionality. New blood vessels are required to remove degradation products that could otherwise accumulate and reach toxic levels. Vascularization is irreplaceable for the nutrient supply of the implants [171]. The generation of new blood vessels is affected by the supplementation of various factors, as described by Jeon et al. [177]. They studied the effect of 3D fibrin gels in microfluidic devices on human mesenchymal stem cells in contact with human endothelial cells by analyzing the creation of microvascular networks via fluorescent microscopy. Fibrin gels supplemented with VEGF, VEGF + angiopoietin 1 (Ang1) or VEGF + transforming growth factor-β1 (TGF-β1) were tested. The addition of Ang1 or TGF-β1 each resulted in increased formation of α-smooth muscle actin, but the addition of TGF-β1 resulted in the generation of non-interconnected microvascular networks, whereas the addition of Ang1 showed the creation of functional networks. Jeon et al. mentioned that the implementation of a microvascular network offers promising potential for the development of advanced, perfusion-capable 3D in vitro models that can be used to test therapeutics and to understand the underlying mechanisms, for example, in tissue regeneration processes. The study was performed with a single-channel microfluidic chip, but Jeon et al. stated that the chip can easily be adapted to high-throughput analysis by using multichannel microfluidic chips.

Another interesting aspect of microfluidic devices is the ability to create defined and closed reaction chambers by hydrodynamic flow focusing [43,121]. Each droplet generated in the process can be viewed as independent, which leads to an almost infinite number of available micro- or nanoreaction chambers. As demonstrated by Fan et al. [178], droplet-based microfluidic devices have the potential to analyze the influence of chemical stimuli on cells. They trapped single cells in a droplet-based microfluidic device and observed them by impedance analysis of the droplet. As a result, they showed differentiation of human bone-marrow-derived MSCs in commercially available differentiation medium, which is indicated by a decrease in the normalized impedance over a 21-day cultivation. Findings are in agreement with the results obtained from alkaline phosphatase activity assay and Alizarin Red S staining. Fan et al. concluded that impedance monitoring can be used to analyze osteogenic differentiation and that it has potential for high-throughput screening based simply on upscaling multiple microelectrodes on a chip. A study by Hayes et al. [179] combined a droplet-based microfluidic system with the frequently used method of real-time quantitative PCR for quantification of differences in gene expression levels. They developed the instrument as a continuous flow system with a fully automated fluidic handling system, droplet generation, distinctive reagent mixing, thermal cycling and an optical detection unit in one device. A higher sample throughput was achieved at a lower cost compared to marketed instruments by combining the two methods. Using robust microdroplets prevents sample-to-sample contamination and reduces the required total volume per sample from 5 to 0.3 µL [179].

Various parameters, such as antibacterial activity, cell attachment, proliferation, gene expression, calcification or inflammatory responses, can be analyzed using the methods discussed. Conclusions can be drawn about the biocompatibility of the analyzed material by considering several parameters simultaneously. According to ISO 10993-1 FDA guidance, risk assessment should “consider the proposed clinical use of the device, including the anatomical location, duration of exposure, and intended use population” [180]. The parameters to be analyzed are, therefore, device-dependent and must be selected and evaluated individually for each medical device. By using high-throughput assays, the number of suitable samples can be limited to a few candidates, which later can be analyzed more accurately and comprehensively to guarantee biocompatibility and safety.

## 5. Conclusions and Perspective

Recent years have seen an increasing demand for implants, particularly bone implants, partly as a result of an aging population. Implants need better functional and antibacterial properties in order to counteract age-related problems—such as reduced healing abilities and high complication rates—and to improve the overall success of treatment in tissue regeneration. Over the past decade, several novel materials have been developed for an optimal bone implant material that is mechanically stable, biodegradable, antimicrobial, biocompatible, osteoinductive and easy to manufacture.

Biologically inert metals have been the standard as bone graft substitutes because of long-term clinical experience and their high mechanical stability [49]. Recent research has focused more on the creation of biodegradable materials such as magnesium alloys, ceramics and polymers. Improvements have been achieved by modifying the materials, for example with nanoparticles to increase antibiotic effects [70,71,72] or cell functionality [73,121,194], with metal ions to take advantage of their functional role in the physiological and cellular environment [54,55,56] or with proteins or RNAs to induce osteosynthesis [16,17,18,19,20]. The use of magnesium alloys shows promising potential as a bone graft substitute [65,195], not only because of its mechanical properties but also because of its physiological ability to induce the migration of osteoblasts to the implantation site as they migrate to environments of high Mg ion concentrations [56]. This leads to a higher number of osteoblast cells at the implant and subsequently to faster bone regeneration. Further modification of magnesium alloys can be achieved by adapting the biological degradation rate to match the bone ingrowth rate using calcium phosphate coatings [195]. Ceramics have been, and still are, a material of interest over the past few decades because of their chemical similarity to natural bone [14]. However, ceramics have the disadvantage of being brittle [84,85] and are therefore not suitable for implants that are highly affected by external forces [59,196]. Nevertheless, ceramics show promising potential as a delivery system for therapeutics, for example as nanocapsules or microspheres incorporated into the scaffold [79,80]. Further research and development of new manufacturing processes will improve the mechanical stability of ceramics while reducing brittleness, leading to the development of a bone implant that resembles natural bone [81]. The last reviewed category of materials, polymers, have attracted the most attention over the last few years due to its enormous potential through modifications. There are various polymers with good biocompatibility and biodegradable properties, which are preferred in the design of new materials [63,174,197,198]. Among the non-biodegradable polymers such as polyethylene (PE), polyethylene terephthalate (PET) or poly (ether ether) ketone (PEEK), there are some modified versions that may qualify as antimicrobial surfaces [60,92,93]. Most research studies, however, have focused on biodegradable polymers as these have the ability to be replaced by natural tissue [11]. There are various biodegradable polymers available, such as polylactic acid (PLA), polycaprolactone (PCL), poly (lactic-*co*-glycolic acid) (PLGA) and chitosan. The number of usable polymers in combination with modifications through copolymerization, polymer blends, generation of special geometries, chemical modification of monomers or combining polymers with inorganic nanocomposites leads to an enormous number of different possible bone grafts [37,38,90]. Although the mentioned categories of materials can be used individually, most research studies have used them in combination to create in composite scaffolds. Combining different materials can overcome the limitations of individual components. A suitable material alone is not enough to meet the patients’ expectations for novel implants. In order to shorten the treatment time and increase the chances of surgery success, the support of implants by mesenchymal stem cell (MSC) therapy appears promising [126,137,138]. For clinical applications, autologous MSCs are the most promising candidates, as the use of allogeneic MSCs can lead to complications such as implant rejection [199]. Seeding autologous MSCs on the implant before implantation can dramatically increase the number of initial stem cells and osteoblasts that induce bone regeneration. Treatment time can be reduced [200] and implant performance can be increased [201] by reducing the time needed for cell proliferation and migration to the implantation site.

The nearly unlimited number of material combinations and modifications results in the creation of many new materials that need to be evaluated. In handling the increasing number of samples, high-throughput screening systems play an essential role as analytics seems to be a bottleneck in the development of new implants [42]. Various approaches to establish high-throughput screening systems are part of research studies and range from semiautomated standard methods for increased throughput [136,167] to microfluidic systems working in nanoliter scale for saving space and reagents [178,179]. All approaches aim at an increased sample throughput in combination with robustness and necessary sensitivity. Semiautomated standard cultivation systems are relatively easy to establish when a liquid handling system is available. However, they are not suitable for analyzing sample numbers in the tens of thousands as the system is based on well plates and therefore the available space for plates in the system limits the sample number. There is a greater potential for high-throughput screenings with a higher number of samples when using microarray or microfluidic devices—especially microfluidic devices based on droplets—as several of these can be operated at the nanoliter scale [179]. Microarray devices have shown to be suitable for analyzing printable material compositions or immobilizable molecules such as proteins or RNA/DNA and are recommended for characterizing libraries [44,202]. Microfluidic devices are recommended when the effect of soluble molecules needs to be analyzed, even though the creation of these devices via standard soft lithography can be costly and time-consuming [185,188]. Creating three-dimensional chip designs using soft lithography is also complicated because chips must be manufactured layer by layer and subsequently bonded together. Recent developments in the field of additive manufacturing have shown the fabrication of three-dimensional microfluidic devices with the required accuracy using fused deposition modeling (FDM) 3D printers [183,184]. The use of 3D printers will make microfluidic device manufacture more affordable, convenient and accessible to a larger number of scientists and industrial users [45,46].

## Figures and Tables

**Table 1:** Combination of keywords used for database creation.

Section	Search Criteria	Number of Hits
	“large bone defect” AND “bone graft substitute”	297 *
Section 2.1	“large bone defect” AND “metal” AND “bone graft substitute”	120 *
Section 2.2	“large bone defect” AND “ceramic” AND “bone graft substitute”	169 *
Section 2.3	“large bone defect” AND “polymer” AND “bone graft substitute”	208 *
Section 2.3	“large bone defect” AND “biodegradable polymer” AND “bone graft substitute”	69 *
Section 2.3	“large bone defect” AND “chitosan” and “bone graft substitute”	85 *
Section 3	Checked hits from Section 2 for “stem cells”	87
Section 4	“in vitro” AND “high throughput” AND “osteogenesis”	795 *

* From sciencedirect.com, checked on 4 November 2020.

**Table 2:** Characteristics and applications of various cell-based high-throughput screening devices.

Device	Advantages	Limits	Applications
Automated cell culture	Based on established methods and protocols Adaptable to different applications	Liquid handling system required Space-consuming instruments Sample number limited by number of wells	Library screening [136,167,170,181]; Enzyme activity screening [169]
Microarray	Screening of large sample libraries Automated printing and imaging of devices Commercially available manufacturing equipment	Costly manufacturing equipment Sample number limited by spotter’s accuracy and chip size Samples need to be immobilized on surfaces or printable	Polymer surfaces, cell attachment [173,175]; Binary polymer blends [174]; Lectin–glycan interaction [164]; Peptide-functionalized hydrogels [44]
Microfluidic—continuous flow mode	Adaptable to different applications Single-cell observations Preparation/mixing and cultivation in one device Coupling with different detection methods: e.g., image-based analysis or mass spectrometry Individual device construction via 3D printer possible [182,183,184]	Chip design complexity increases with sample number Sample number limited by chip size and accuracy for creating enclosed chambers [185]	Single-cell analysis [43,186]; Vascularization [177]; Neuromuscular circuits [187]; Evaluation of nanobiomaterials [13]
Droplet-based microfluidic	Sample and reagent consumption in nanoliter range Single-cell observations Preparation/mixing and cultivation in one device Coupling with different detection methods: e.g., image-based analysis, mass spectrometry or capillary electrophoresis	Complex statistics needed to sort analysis data Difficult to perform long-term culture	Droplet generation [188,189]; Mixing inside droplet [160,190]; Droplet-based microfluidic PCR [179]; Single-cell analysis [178,191]; Metagenomic library screening [192]; Imaging-based droplet analysis [193]

## Data Availability

Data sharing not applicable. No new data were created or analyzed in this study. Data sharing is not applicable to this article.

## Abbreviations

µ-PET	µ-Positron emission tomography
β-TCP	β-Tricalcium phosphate
ALP	Alkaline phosphatase
Ang1	Angiopoietin 1
BMP	Bone morphogenetic protein
FDM	Fused deposition modeling
HA	Hydroxyapatite
HACC	Hydroxypropyltrimethyl ammonium chloride chitosan
Mg	Magnesium
MSC	Mesenchymal stem cell
PCL	Polycaprolactone
PE	Polyethylene
PEEK	Poly (ether ether) ketone
PET	Polyethylene terephthalate
PGA	Polyglycolic acid
PLA	Polylactic acid
PLGA	Poly (lactic-co-glycolic acid)
qRT-PCR	Quantitative reverse transcription polymerase chain reaction
Sr	Strontium
TGF-β1	Transforming growth factor-β1
VEGF	Vascular endothelial growth factor
WHO	World Health Organization
